# CD57 Expression and Cytokine Production by T Cells in Lesional and Unaffected Skin from Patients with Psoriasis

**DOI:** 10.1371/journal.pone.0052144

**Published:** 2013-02-28

**Authors:** Mariana D. Batista, Camilla Tincati, Jeffrey M. Milush, Emily L. Ho, Lishomwa C. Ndhlovu, Vanessa A. York, Esper G. Kallas, Jorge Kalil, Sheila M. Keating, Philip J. Norris, David Chang, Patrick Unemori, Kieron S. Leslie, Toby Maurer, Wilson Liao, Douglas F. Nixon

**Affiliations:** 1 Division of Experimental Medicine, Department of Medicine, University of California San Francisco, San Francisco, California, United States of America; 2 Division of Clinical Immunology and Allergy, School of Medicine, University of São Paulo, São Paulo, Brazil; 3 Instituto de Investigação em Imunologia, University of São Paulo, São Paulo, Brazil; 4 Blood Systems Research Institute, San Francisco, California, United States of America; 5 Department of Laboratory Medicine, University of California San Francisco, San Francisco, California, United States of America; 6 Department of Medicine, University of California San Francisco, San Francisco, California, United States of America; 7 California Pacific Medical Center - Davies Campus, San Francisco, California, United States of America; 8 Department of Dermatology, University of California San Francisco, San Francisco, California, United States of America; Centro de Pesquisa Rene Rachou/Fundação Oswaldo Cruz (Fiocruz-Minas), Brazil

## Abstract

**Background:**

The immunopathogenic mechanisms leading to psoriasis remain unresolved. CD57 is a marker of replicative inability and immunosenescence on CD8+ T cells and the proportion of CD57 expressing CD8+ T cells is increased in a number of inflammatory conditions.

**Methodology:**

We examined the expression of CD57 on T cells in the skin of patients affected with psoriasis, comparing lesional and unaffected skin. We also assessed functionality of the T cells by evaluating the secretion of several inflammatory cytokines (IL-17A, IFN-gamma, IL-2, IL-33, TNF-alpha, IL-21, IL-22, and IL-27), from cell-sorted purified CD4+ and CD8+ T cells isolated from lesional and unaffected skin biopsies of psoriasis patients.

**Principal Findings:**

We observed that the frequency of CD57+CD4+ and CD57+CD8+ T cells was significantly higher in unaffected skin of psoriasis patients compared to lesional skin. Sorted CD4+ T cells from psoriatic lesional skin produced higher levels of IL-17A, IL-22, and IFN-gamma compared to unaffected skin, while sorted CD8+ T cells from lesional skin produced higher levels of IL-17, IL-22, IFN-gamma, TNF-alpha, and IL-2 compared to unaffected skin.

**Conclusions/Significance:**

These findings suggest that T cells in unaffected skin from psoriasis patients exhibit a phenotype compatible with replicative inability. As they have a lower replicative capacity, CD57+ T cells are less frequent in lesional tissue due to the high cellular turnover.

## Introduction

Psoriasis is an inflammatory skin disease where immunologic imbalance and altered keratinocyte differentiation lead to hyperproliferation of the skin [1]. Although psoriasis was initially classified as a Th-1-polarized disease, a clear role for CD4+ T cells that produce IL-17A and IL-22 (Th-17 and Th-22 cells) has been established in recent years, primarily at lesional sites, but also in the blood [2], [3], [4]. The inflammatory milieu is the key determinant for plaque development and maintenance, and each cell type involved in the process has its own characteristic signature cytokines. In psoriasis, IFN-gamma, the prototype Th-1 cytokine, interplays with IL-2, TNF-alpha, IL-17, and IL-22 to contribute to inflammation and altered differentiation [2], [5], [6].

Much debate exists regarding the relative contribution of CD8+ T cells to cytokine production in psoriasis, as CD8+ T cells may also produce IL-17 and IL-22 [7], [8]. CD57 is a marker of replicative inability on T cells. CD57+CD8+ T cells expand in a number of conditions of chronic immune activation, such as viral infections [9], inflammatory diseases, including rheumatoid arthritis and Wegener granulomatosis [10], [11], and malignancies [12]. CD57+CD8+ T cells can also be expanded after physical stress [13] and in aging [14]. Although these cells exhibit limited proliferative and survival abilities, they nonetheless manifest high cytotoxic properties, being destined to migrate to non-lymphoid tissues without further cycling [12], [15], [16]. We sought to investigate the role of CD57 expression on T cells in lesional and non-lesional unaffected skin of psoriasis patients.

## Results

### Patient demographics

Twenty patients with psoriasis were included in this study, 11 males and 9 females. Severity was distributed as follows: mild (n = 10), moderate (n = 6), and severe (n = 4). The median age was 51 years (inter-quartile range 38–56 years). The most common predisposing factors listed were stress, skin injury, and lack of sun exposure during winter.

### Increased CD57 expression on CD4+ and CD8+ T cells in unaffected skin of psoriatic patients

**Figure 1:**
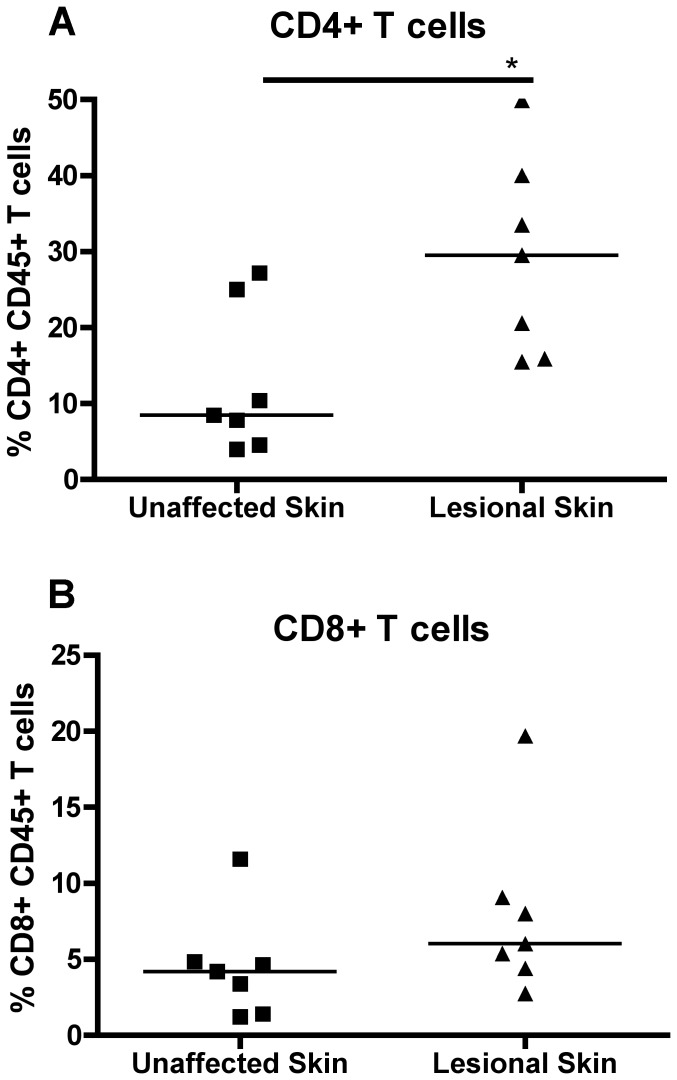
T cell distribution in skin and PBMC of psoriasis patients. Frequency of (A) CD4^+^ T cells and (B) CD8^+^ T cells in the skin (lesional and unaffected) from psoriasis patients (n = 7). * = p<0.05.

We first determined whether the CD4+ or CD8+ T cell distribution was altered in unaffected skin compared to lesional skin of psoriasis patients. CD45+ leukocytes in skin samples (psoriatic lesions and non-lesional) were assessed by flow cytometry. We observed a significantly higher percentage of CD4+ T cells in lesional skin compared to unaffected skin (Figure 1A). Although there was a trend towards higher percentages of CD8+ T cells in lesional skin, the difference was not significant (Figure 1B).

**Figure 2:**
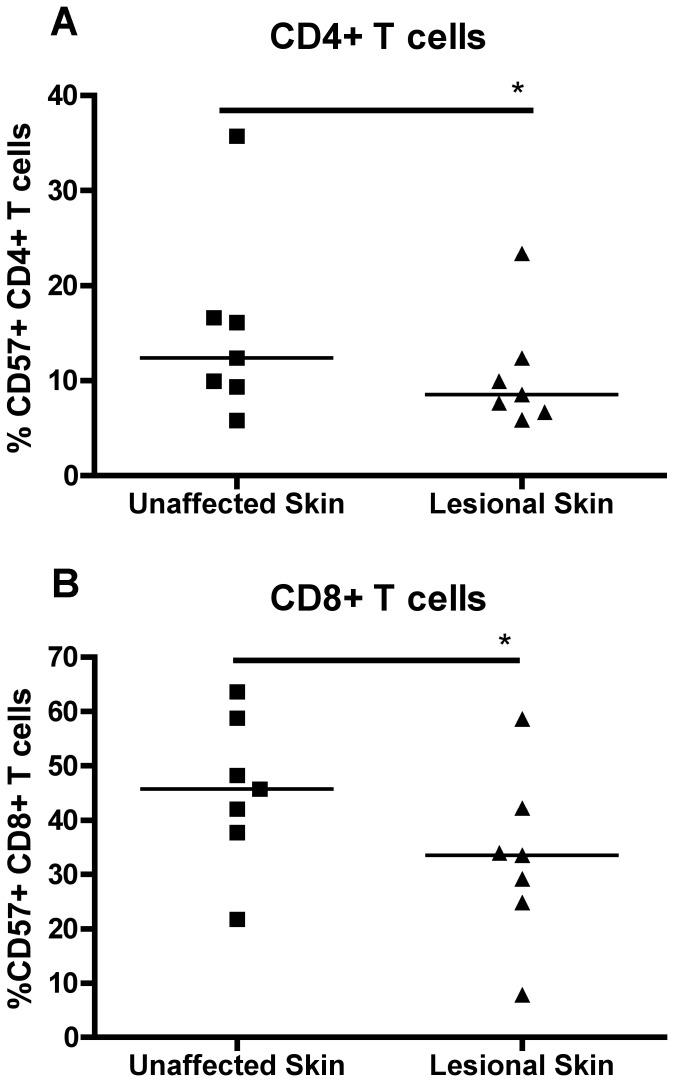
CD57 expression on T cells of psoriasis patients. CD57 expression on (A) CD4^+^ T cells and (B) CD8^+^ T cells from the skin of psoriasis patients (n = 7). * = p<0.05.

To determine whether T cells were terminally differentiated, we examined the frequency of CD57+ T cells in the skin (Figure 2A and B). Interestingly, the frequency of CD57+CD4+ and CD57+CD8+ T cells was significantly higher in unaffected skin of psoriasis patients compared to lesional skin (Figures 2A and 2B). CD57 expression in skin was not correlated with the subject's age (data not shown).

### Sorted CD4+ and CD8+ T cells from lesional skin produce higher levels of cytokines than unaffected skin

Several cytokines have been known to play in role in psoriasis, however the secretion at different sites and the cell type producing them remains unknown. We therefore performed a selected cytokines Multiplex assay to measure key inflammatory mediators IL-17A, IFN-gamma, IL-2, IL-33, TNF-alpha, IL-21, IL-22, and IL-27.

**Figure 3:**
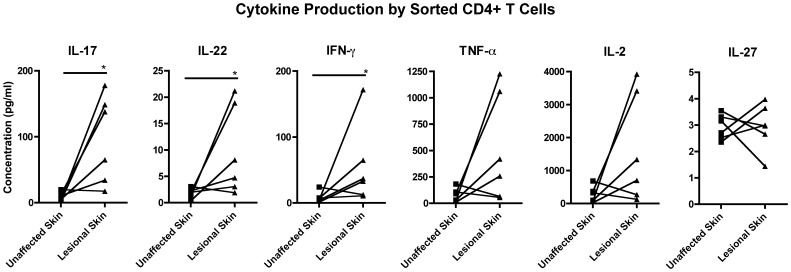
Sorted CD4+ T cell cytokine production. Cytokine production by sorted CD4+ T cells from unaffected and lesional skin from psoriasis patients, with stimulation with PMA-ionomycin. Comparative chart representing IL-17A, IL-22, IL-2, IFN-gamma, TNF-alpha and IL-27. * = p<0.05.

While unstimulated samples from all compartments did not seem to produce significant cytokine levels, we observed that lesional skin CD4+ T cells stimulated with PMA-ionomycin produced higher levels of IL-17A, IL-22, and IFN-gamma in relation to unaffected skin from the same patients (Figure 3). There was a trend to higher production of TNF-alpha and IL-2 by sorted CD4+ T cells from lesional skin, while no difference was observed for IL-27 levels (Figure 3).

**Figure 4:**
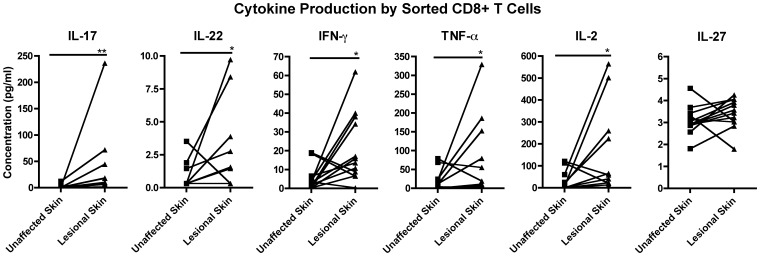
Sorted CD8+ T cell cytokine production. Cytokine production by sorted CD8+ T cells from unaffected and lesional skin from psoriasis patients, with stimulation with PMA-ionomycin. Comparative chart representing IL-17A, IL-22, IL-2, IFN-gamma, TNF-alpha and IL-27. * = p<0.05, ** = p<0.01.

For CD8+ T cells, we observed that the production of IL-17A, IL-22, IFN-gamma, TNF-alpha, and IL-2 were higher in lesional skin than unaffected skin of psoriasis patients (Figure 4).

Despite the fact that IL-21 and IL-33 have been implicated in psoriasis pathogenesis [17], [18], [19], we could not find any difference in the production of these cytokines between lesional and unaffected skin of psoriasis patients (data not shown).

### IL-22 levels produced by CD4+ T cells were correlated to other cytokines

**Figure 5:**
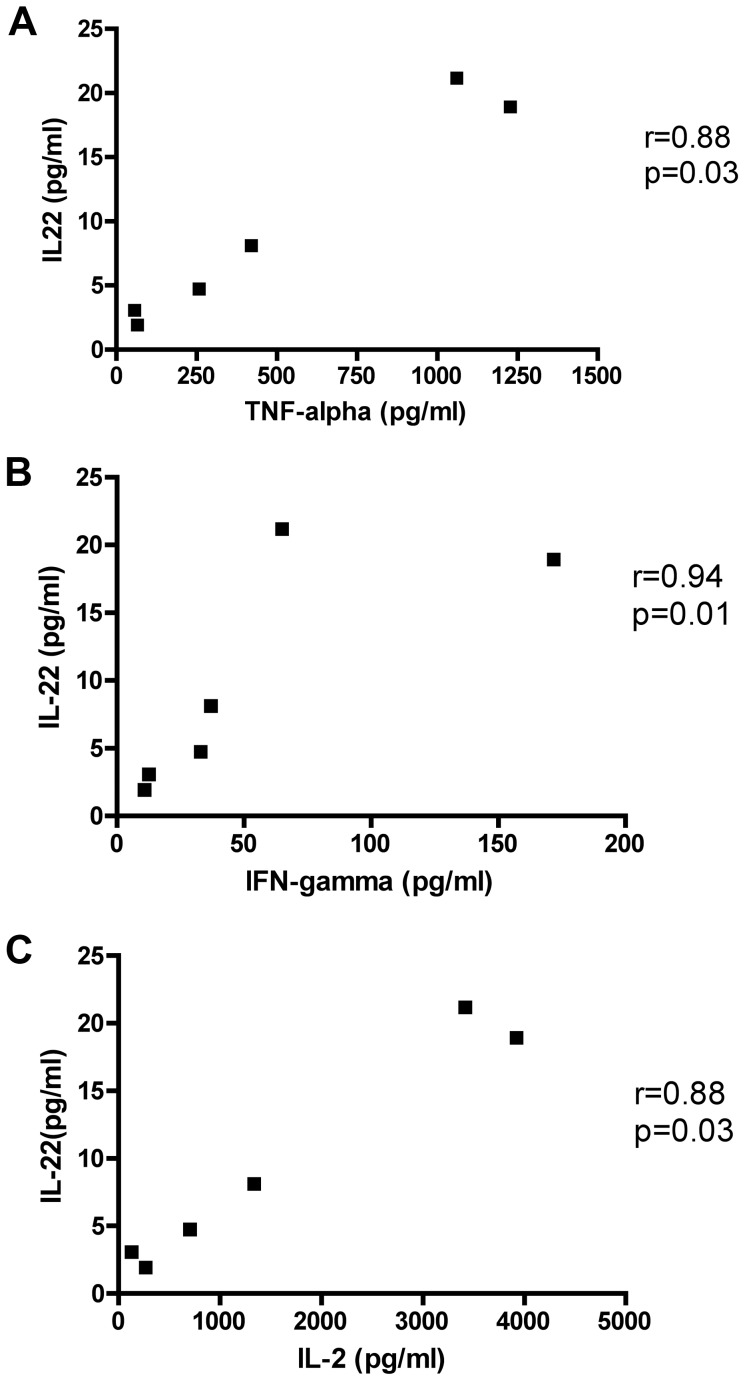
IL-22 levels correlate to other cytokines. Correlation of IL-22 levels produced by lesional skin CD4+ T cell with (A) TNF-alpha (B) IFN-gamma (C) IL-2.

We next assessed whether the levels of IL-17A or IL-22 produced by lesional skin CD4+ or CD8+ T cells were correlated to the other cytokines that were tested. Only the levels of IL-22 produced by CD4+ T cells isolated from lesional skin were correlated to TNF-alpha, IFN-gamma, and IL-2 (Figure 5). No other associations were found.

## Discussion

CD57 expression on CD8+ T cells determines lack of proliferation ability, associated with short telomeres, defining replicative senescence that occurs in conditions of chronic immune activation, such as HIV-1 infection [16]. CD57+CD8+ T cells exhibit up-regulated expression of cytotoxicity genes, such as perforin, granulolysin, and granzyme B, indicating increased cytotoxic ability, and a higher IFN-gamma and TNF-alpha production upon TCR stimulation [15], [20]. On CD4+ T cells, CD57 expression has also been associated with decreased proliferation capacity, and affects CD4+ T cell function, being associated with higher IFN-gamma but lower IL-2 production [21]. Whether CD57+CD4+ and CD57+CD8+ T cells can contribute to psoriasis immunopathogenesis remains poorly understood. One previous study has demonstrated by immunohistochemistry the presence of CD57+ cells in psoriasis patients, showing higher numbers in involved epidermis and papillary dermis compared to uninvolved skin, while in reticular dermis, uninvolved skin exhibited higher numbers of CD57+ cells compared to normal control skin [22].

CD57+CD8+ T cells exhibit a phenotype compatible with increased ability to migrate to tissues without further cycling [12]. They express higher levels of CX3CR1 than CD57−CD8+ T cells, which can dictate their migration to tissues [15]. Interestingly, 2 polymorphisms in the CX3CR1 gene were found to be associated with psoriasis [23]. One of these was a coding polymorphism with higher frequency in healthy controls compared to cases that is thought to impair the ability of CX3CR1 to adhere to its ligand CX3CL1 [23]. These genetic data support the notion that recruitment of CD57+ lymphocytes into the skin via the CX3CR1-CX3CL1 axis may be important in the pathogenesis of psoriasis.

In this study, we found higher CD57 expression on CD4+ and CD8+ T cells in unaffected skin of psoriasis patients compared to lesional skin. In recent years, much attention has been given to non-lesional unaffected skin of psoriasis patients, where quiescent auto-reactive T cells named skin-resident T cells have been demonstrated [24]. CD57 is a marker of chronic antigenic stimulation. We could hypothesize that some of the quiescent T cells present in non-lesional sites express CD57 as a result of previous antigenic stimulation. We do not know whether the site of unaffected skin biopsy has previously been a lesional site. The persistence of a population of CD8+ T cells in the dermis of resolved psoriasis lesions after treatment has been demonstrated, and points to the possibility of lesional memory [25], [26]. Alternatively, as CD57 expression on T cells is associated with a decreased replicative capacity, the high cellular turnover at lesional sites could result in the lower survival of CD57+ T cells, leading to the finding of lower CD57 expression in psoriasis lesions.

Unlike atopic dermatitis, where clear differences have been shown between unaffected skin and normal skin from healthy controls, in psoriasis unaffected skin has been considered largely similar to normal skin [27]. However, differences in gene expression between uninvolved psoriasis skin and normal control skin have also been shown [28]. We have found higher CD57 expression in unaffected psoriasis skin compared to lesional skin. CD57 expression on T cells has not been described in normal control skin. We believe that the finding of higher CD57 expression is specific to uninvolved psoriasis skin, and is not a consequence of similarities between uninvolved psoriasis skin and normal control skin.

Th-17 cells constitute a subset of CD4+ T cells that have the distinct characteristics of being stimulated by IL-23, and producing IL-17A [29]. IL-17A has been implicated as an important cytokine in the pathogenesis of psoriasis. IL-17 mRNA levels are higher in lesional skin in psoriasis than in normal skin from healthy controls [3], [30]. The presence of IL-17A in lesional tissue leads to the release of multiple inflammatory cytokines by keratinocytes, including TNF-alpha, IL-1, and IL-6 [3], [31]. IL-17A can also induce angiogenesis and the production of antibacterial peptides [32]. In addition, IL-17A induces keratinocyte expression of chemokine genes that lead to neutrophil migration, thus contributing to overall inflammation [4]. That is in contrast with the effects of IFN-gamma, which induces preferential expression of CXCL9, CXCL10, and CXCL11, that bind to activated T cells containing CXCR3 [4].

IL-22, another important cytokine for psoriasis pathogenesis, can be produced by CD4+ T and CD8+ T cells [8], [33]. IL-22 induces multiple anti-apoptotic and proliferative pathways through the Stat3 cascade and has the capacity of inducing keratinocyte proliferation through the down-regulation of keratinocyte differentiation genes, such as keratin 1 and fillagrin [34], [35]. IL-22 induces anti-microbial proteins such as beta-defensins and IL-22 mRNA expression is increased in psoriasis lesions compared to normal skin [36], [37].

In this study, we were able to compare the relative production of IL-17 and IL-22, as well as IFN-gamma, IL-2, TNF-alpha, and IL-27, in lesional and unaffected skin on the same individuals. We found that CD4+ and CD8+ T cells isolated from lesional skin have a higher ability to produce IL-17A than their counterparts isolated from unaffected skin. We could hypothesize that T cells in lesional skin have acquired the ability to secrete this cytokine due to the local inflammatory environment or locally expressed antigens. IL-22 production by CD4+ T cells isolated from lesional skin was also higher than unaffected skin. They could correspond to Th-17 cells or Th-22 cells. We could also demonstrate IL-22 production by sorted CD8+ T cells from lesional skin in low amounts, in higher levels than unaffected skin. Interestingly, IL-22 production by CD4+ T cells isolated from lesional skin correlated with TNF-alpha, IFN-gamma, and IL-2. CD8+ T cells isolated from lesional skin exhibited a mixed cytokine profile between Th-17 and Th-1, as higher production of IFN-gamma, TNF-alpha, and IL-2 was also observed in lesional skin.

In conclusion, we were able to assess phenotypic characteristics and cytokine production by CD4+ and CD8+ T cells from lesional and unaffected skin from the same patients. We demonstrated that there are phenotypic and functional differences in T cells in different sites in the same individuals. Further studies are needed to elucidate the importance of unaffected tissue in psoriasis.

## Materials and Methods

### Patient Selection

Twenty psoriasis patients were recruited for this study. Patients provided informed consent and the study was approved by the UCSF Institutional Review Board, according to the declaration of Helsinki. Disease ranged from mild to severe and most patients had a long history of psoriasis (median 12 years). Severity was assessed based on affected body surface area (BSA), as follows: mild - less than 5% BSA, moderate - 5–30% BSA, severe - more than 30% BSA. Subjects included in the study were recruited during their first visit to the Dermatology Clinic at San Francisco General Hospital, and had not used topical or systemic therapy at least 2 months prior to sample collection.

Patients were divided into 2 groups. For the phenotypic portion of the study, 7 psoriasis patients were included. For the functional part of the study (cell sorting and cytokine production), 13 patients were included. Lesional and unaffected skin samples were available for all patients. One patient was excluded from the study due to low post-sorting purity levels.

### Skin sample preparation

Skin samples were collected as 4-mm punch biopsies, one from an active psoriatic lesion and another from unaffected skin, at least 5 cm away from an active lesion. The samples were incubated for 15 minutes at 37°C and then overnight at 4°C in 1 mg/ml collagenase/dispase (Roche Diagnostics, Indianapolis, IN, USA) and 10 units/ml recombinant DNAse I (Roche Diagnostics, Indianapolis, IN, USA). Epidermis and dermis were both cultured separately for 48 hours at 37°C in RPMI-1640 supplemented with 10% pooled human serum (Gemini Bio Products, Sacramento, CA, USA), 1% penicillin and streptomycin, and 1 mol/L HEPES buffer (Invitrogen, Carlsbad, CA, USA) in 6-well culture plates. Single cell suspensions were obtained after rinsing through a 70 µm cell strainer (BD, San Jose, CA, USA), and cells isolated from epidermis and dermis were subsequently combined to produce sufficient cell numbers for the subsequent experiments.

### Flow cytometry

A multi-parameter flow cytometry analysis was performed using an LSR II flow cytometer (BD Biosciences). Cells were first Fc receptor blocked using 10 µg/ml human IgG (Sigma Aldrich, S. Louis, MI, USA) for 20 minutes on ice. Cells were then stained for 30 minutes on ice with fluorophore-labeled antibodies, washed with FACS buffer, and fixed with 2% paraformaldehyde (Touisimis, Rockville, MD) in PBS. The data files were analyzed using FlowJo Software version 8.8.6 (Tree Star, San Carlos, CA, USA). The gating strategy is demonstrated in Figure S1.

### CD4+ and CD8+ T cell sorting

Cells were stained using fluorescein isothiocyanate (FITC)-conjugated anti-CD45 (eBioscience, San Diego, CA, USA), phycoerythrin-Texas Red (ECD)-conjugated anti-CD3 (Beckman Coulter, Brea, CA, USA), Alexa700-conjugated anti-CD4 (BD Biosciences, San Jose, CA, USA), allophycocyanin-Cy7 (APC-Cy7)-conjugated anti-CD8 (Biolegend, San Diego, CA, USA), and Amine Aqua (AARD) for live/dead discrimination (Invitrogen, Carlsbad, CA, USA). Freshly isolated PBMCs and skin cells were incubated with fluorochrome-conjugated antibodies and AARD antibodies for 30 minutes on ice. Cells were then washed twice with FACS buffer, and sorted on a FACS Aria flow cytometer (BD Biosciences, San Jose, CA, USA). During the first experiment (n = 6), only CD8+ T cells were sorted, using a doublet discrimination gating followed by gating on CD45+ cells and CD3+CD8+ T cells. On the second experiment (n = 7), CD4+ and CD8+ T cells were sorted using a doublet discrimination gating followed by gating on CD45+ cells and finally on CD3+CD4+ and CD3+CD8+ T cells. The purities of all sorts were greater than 95%. One patient was excluded due to a 75% post-sorting purity level.

### T cell cultures

Sorted CD4+ and CD8+ T cells isolated from lesional and unaffected skin and PBMCs from psoriatic patients were cultured for 8 hours at 37°C and 5% CO_2_ with or without PMA (50 ng/ml) and ionomycin (500 ng/ml) stimulation (Sigma-Aldrich, St. Louis, MO, USA) in a 96-well U-bottom plate. The plate was centrifuged, the culture supernatant carefully removed, the cells were washed with PBS, and fresh culture media containing IL-2 without PMA and ionomycin was added. The cells were then placed back in culture at 37°C and 5% CO_2_ for 7 days. On day 7 the plate was centrifuged and cell-free supernatants were frozen at −20°C for subsequent use in Luminex analyses.

### Luminex assays

Production of IL-17A, IFN-gamma, IL-2, IL-33, TNF-alpha, IL-21, IL-22 and IL-27 were assayed using Multiplex cytokine arrays (Biolegend, San Diego, CA, USA) following the manufacturer's protocols. Samples were acquired on a Labscan 200 analyzer (Luminex, Austin, TX, USA) using Bio-Plex manager 6.0 software (Bio-Rad, Hercules, CA, USA). It should be noted that the media for T-cell culture contained IL-2, so the comparative analyses between groups are possible, though the analyte concentrations obtained reflect the IL-2 added in the media.

### Statistical Analyses

The statistical analyses were performed using Prism Software version 4.0a (GraphPad, La Jolla, CA, USA). Groups were compared using the Wilcoxon paired non-parametric test with a minimum significance value of p = 0.05.

## Supporting Information

Figure S1 Gating strategy representing lesional skin sample.
(TIFF)
