# Supplementary figures and images for: CD57 Expression and Cytokine Production by T Cells in Lesional and Unaffected Skin from Patients with Psoriasis
Source: PLoS One. 2013 Feb 28;8(2):e52144. doi: 10.1371/journal.pone.0052144 (PMC3585296; doi:10.1371/journal.pone.0052144)

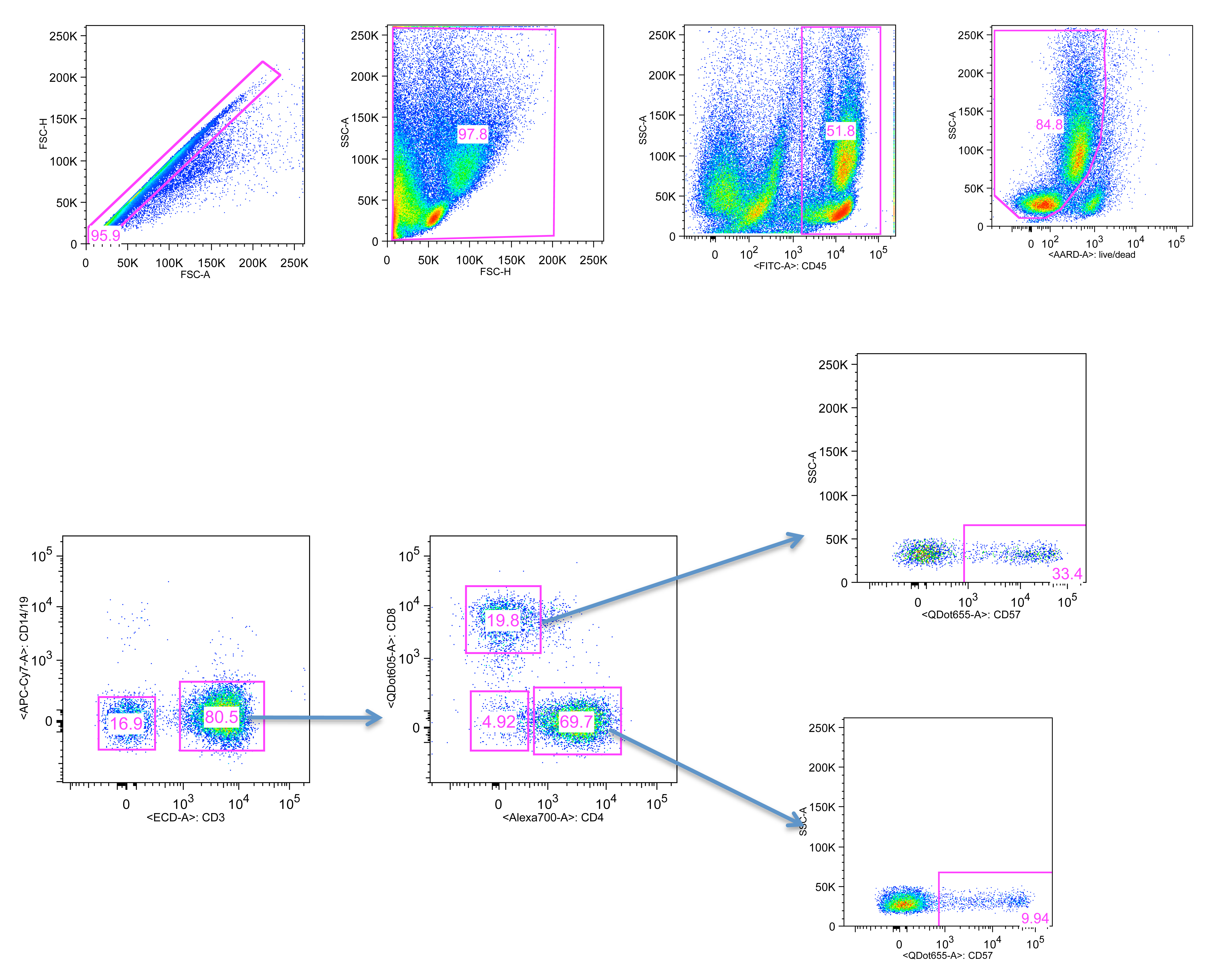

Supplement: Figure S1 — Gating strategy representing lesional skin sample. (TIFF) [file pone.0052144.s001.tiff]
